# Clinical outcomes and risk factors for COVID-19 among migrant populations in high-income countries: A systematic review

**DOI:** 10.1016/j.jmh.2021.100041

**Published:** 2021-04-22

**Authors:** Sally E Hayward, Anna Deal, Cherie Cheng, Alison Crawshaw, Miriam Orcutt, Tushna F Vandrevala, Marie Norredam, Manuel Carballo, Yusuf Ciftci, Ana Requena-Méndez, Christina Greenaway, Jessica Carter, Felicity Knights, Anushka Mehrotra, Farah Seedat, Kayvan Bozorgmehr, Apostolos Veizis, Ines Campos-Matos, Fatima Wurie, Martin McKee, Bernadette Kumar, Sally Hargreaves, on behalf of the ESCMID Study Group for Infections in Travellers and Migrants (ESGITM)

**Affiliations:** a Institute for Infection and Immunity, St George's University of London, London, UK; b Institute for Global Health, University College London, London, UK; c Faculty of Business and Social Sciences, Kingston University, London, UK; d Danish Research Centre for Migration, Ethnicity and Health, University of Copenhagen; Department of Infectious Diseases at Copenhagen University Hospital, Amager and Hvidovre, Copenhagen, Denmark; e International Centre for Migration, Health, and Development, Geneva, Switzerland; f Doctors of the World UK, London, UK; g Department of Medicine, Karolinska Insitutet, Solna, Sweden; and Barcelona Insitute for Global Health (ISGlobal), University of Barcelona, Barcelona, Spain; h Department of Medicine, McGill University, Montreal, Canada; i Department of Population Medicine and Health and Health Services Research, School of Public Health, Bielefeld University, Bielefeld, Germany; Section for Health Equity Studies & Migration, Heidelberg University Hospital, Heidelberg, Germany; j Medecins Sans Frontieres Greece, Athens, Greece; k Public Health England, London, UK; l Public Health England; and UCL Collaborative Centre for Inclusion Health, London, UK; m Public Health England; and UCL Research Department of Epidemiology and Public Health, London, UK; n Norwegian Institute of Public Health, Oslo, Norway; o Faculty of Public Health and Policy, London School of Hygiene and Tropical Medicine, London, UK

## Abstract

**Background:**

Migrants in high-income countries may be at increased risk of COVID-19 due to their health and social circumstances, yet the extent to which they are affected and their predisposing risk factors are not clearly understood. We did a systematic review to assess clinical outcomes of COVID-19 in migrant populations, indirect health and social impacts, and to determine key risk factors.

**Methods:**

We did a systematic review following PRISMA guidelines (PROSPERO CRD42020222135). We searched multiple databases to 18/11/2020 for peer-reviewed and grey literature on migrants (foreign-born) and COVID-19 in 82 high-income countries. We used our international networks to source national datasets and grey literature. Data were extracted on primary outcomes (cases, hospitalisations, deaths) and we evaluated secondary outcomes on indirect health and social impacts and risk factors using narrative synthesis.

**Results:**

3016 data sources were screened with 158 from 15 countries included in the analysis (35 data sources for primary outcomes: cases [21], hospitalisations [4]; deaths [15]; 123 for secondary outcomes). We found that migrants are at increased risk of infection and are disproportionately represented among COVID-19 cases. Available datasets suggest a similarly disproportionate representation of migrants in reported COVID-19 deaths, as well as increased all-cause mortality in migrants in some countries in 2020. Undocumented migrants, migrant health and care workers, and migrants housed in camps have been especially affected. Migrants experience risk factors including high-risk occupations, overcrowded accommodation, and barriers to healthcare including inadequate information, language barriers, and reduced entitlement.

**Conclusions:**

Migrants in high-income countries are at high risk of exposure to, and infection with, COVID-19. These data are of immediate relevance to national public health and policy responses to the pandemic. Robust data on testing uptake and clinical outcomes in migrants, and barriers and facilitators to COVID-19 vaccination, are urgently needed, alongside strengthening engagement with diverse migrant groups.

## 1 Introduction

The COVID-19 pandemic has highlighted the vast ethnic, social, economic and cultural diversity that has come to characterise contemporary high-income countries (HICs), and has served as a reminder of the growing rate of population movement between, as well as within, countries and the new public health opportunities and challenges this is presenting. One of these challenges is the scale of health and social disparities associated with this diversity, with profound consequences for some ethnic minority groups (Greenaway et al., 2020). Data from several countries have revealed a much greater risk of infection and adverse outcomes from COVID-19 among Black, Asian, and Minority Ethnic [BAME] groups, South/East Asian, Black Americans, Hispanics, Latinos, racialised groups, people of colour, and indigenous groups compared to the native white population in the same countries (Sze and Nevill, 2020). These adverse outcomes are likely the result of a complex interaction of socioeconomic disadvantage influencing exposure to SARS-CoV-2 and underlying health status, that predisposes to severe illness (Public Health England 2020, Mathur et al., 2020), leading to calls to address the root causes of these inequalities now and in the future.

Although a picture is emerging, there is not yet a comprehensive overview of the extent to which migrants (defined as foreign-born) – including refugees, asylum seekers, labour migrants, and undocumented migrants living temporarily or permanently in different HICs – have been impacted by COVID-19, and their specific risk factors. Prior to the COVID-19 pandemic, global migration was at its highest level on record, with 1 billion people on the move around the world, and with HICs receiving unprecedented numbers of people seeking human security either through political asylum and/or work opportunities (Abubakar and Devakumar, 2018). Most of the relatively few health datasets with information on ethnicity currently used to monitor COVID-19 reflect what information is already recorded by healthcare systems (which is highly variable across countries and regions). For the most part these fail to capture migration status, combining those born in the host countries to families that may have been in the country for several generations with more recent migrants, thus failing to reflect the health dynamics of contemporary migration. Although more recently arrived migrants predominantly from low- and middle-income countries, are typically considered to be young and healthy on arrival (Aldridge and Nellums, 2018), and may share many of the characteristics of “older” generation ethnic minorities and their offspring, they may also present a unique spectrum of health and social risk factors for COVID-19 exposure and infection that to date has been poorly defined. In many countries, migrants make up a significant proportion of front-line workers who may have a greater exposure to COVID-19, in sectors witnessing a disproportionate impact of COVID-19 infections (Nicholson and Alulema, 2020). There are, in addition, tens of thousands of migrants in HICs who are being housed in camps, detention centres, and labour dormitories or compounds, all of which are considered high-risk environments for COVID-19. Recent analyses suggest that countries and regions with large migrant populations (including US, Italy, Spain, France, and the UK) should ensure they are better considered in public health responses (Guadagno, 2020, Migration Data Portal 2020).

In order to develop a more targeted and inclusive public health response a better understanding of the impact that COVID-19 is having specifically on migrant populations is critically needed. We therefore did a systematic review to explore and assess what is currently known about clinical outcomes of COVID-19 (cases, hospitalisations, deaths), indirect health and social impacts, and to identify key risk factors and vulnerabilities in migrant populations.

## 2 Methods

### 2.1 Search strategy

We undertook a systematic review in line with the Preferred Reporting Items for Systematic Reviews and Meta-Analyses (PRISMA) guidelines (Moher et al., 2009), and registered with PROSPERO (CRD42020222135). We searched the following databases: Embase, Web of Science, Oxford Academic Journals, PubMed NIH, Clinical Trials, China CDC MMWR, CDC reports, ProQuest Central (Proquest), CINAHL, Africa Wide Information (Ebsco), Scopus, PsycInfo, CAB Abstracts, Global Health, J Stage, Science Direct, Wiley Online Journals, JAMA Network, British Medical Journal, Mary Ann Liebert, New England Journal of Medicine, Sage Publications, Taylor and Francis Online, Springer Link, Biomed Central, MDPI, ASM, PLOS, The Lancet, Cell Press, and pre-print sites chemRxiv, SSRNbioRxiv, and medRxiv facilitated through the WHO Global Research on COVID-19 database from inception to 18/11/2020 (https://search.bvsalud.org/global-literature-on-novel-coronavirus-2019-ncov/). The latter is a daily-updated, multilingual resource of all the global literature (peer-reviewed literature, pre-prints and grey literature) pertaining to COVID-19. We used a broad search strategy encompassing terms related to ethnicity and migrants, to source specific information pertaining to migrants (Appendix 1).

Records were imported into EndNote, and duplicates deleted. Title/abstract and full-text screening were carried out by two reviewers using Rayyan QCRI (Ouzzani et al., 2016). A snowballing method was used to follow up potentially relevant articles cited in included papers. Grey literature sources were also hand-searched. Our international networks were used to directly engage migrant health experts in key countries, who were specifically approached to source country-level public health data (via the Ministry of Health and public health statistics) and other grey literature.

### 2.2 Selection criteria and primary/secondary outcomes

We included any data pertaining to our selected primary and secondary outcomes on migrant populations from 82 World Bank HICs (countries listed in Appendix 2). Migrants were defined as foreign-born individuals, born outside of the country in which they are resident. Primary outcomes were clinical outcomes of COVID-19 in migrant populations (cases, hospitalisation, deaths). Secondary outcomes included indirect health and social impacts, and risk factors and vulnerabilities (co-morbidities, health behaviours and systemic factors, social and cultural factors, and occupation).

No restrictions were imposed on study design because our preliminary scoping review revealed that in this rapidly evolving field important data were often embedded into letters, editorials, and grey literature as well as primary research studies and national statistics. We imposed no language restrictions and information was translated where required. Studies were excluded if it was not possible to determine whether individual(s) in the population studied were migrants, based on the stated criteria, and where data were collected outside of the countries listed or did not directly relate to COVID-19 outcomes, impacts and risk factors. We excluded all mass media reports.

### 2.3 Data extraction, critical appraisal and synthesis

Abstracts were screened and data were extracted in duplicate at each stage, involving three researchers (CC, SEH, SH). Records and data were managed through EndNote and Excel databases prepared by the principal reviewers. The quality of studies was assessed by two reviewers (AD, CC), using Joanna Briggs Institute critical appraisal tools (checklists for cohort studies, qualitative research, prevalence studies, cross-sectional studies, case series or text and opinion checklists, as appropriate for the individual study design) (Joanna Briggs Institute 2020). Quality scores were calculated as a total out of the maximum number of applicable questions and converted into percentages. Studies with a score of 80–100% were considered high quality, 60–79% medium quality and 0–59% low quality. Data sources were not excluded based on study quality, but information on quality contributed to the meta-synthesis and discussion. Only original research was appraised for both primary and secondary outcomes, as the appraisal tools are specific for study designs and thus are not applicable to sources such as commentaries. Critical appraisals were only performed for literature in English, French or Spanish, due to the language restrictions of the critical appraisal team.

For the primary outcomes we included only primary data sources; the heterogeneity of study designs and populations precluded meta-analysis. For the secondary outcomes we included primary data and data from other sources, which was collated and assessed using narrative synthesis.

### 2.4 Patient and public involvement

The authorship of this paper includes a migrant representative member of St George's University of London Migrant Health Project Advisory Group, as well as several professionals working directly with migrants to implement COVID-19-related interventions. These individuals have been involved in all stages of this research.

## 3 Results

**Fig. 1:**
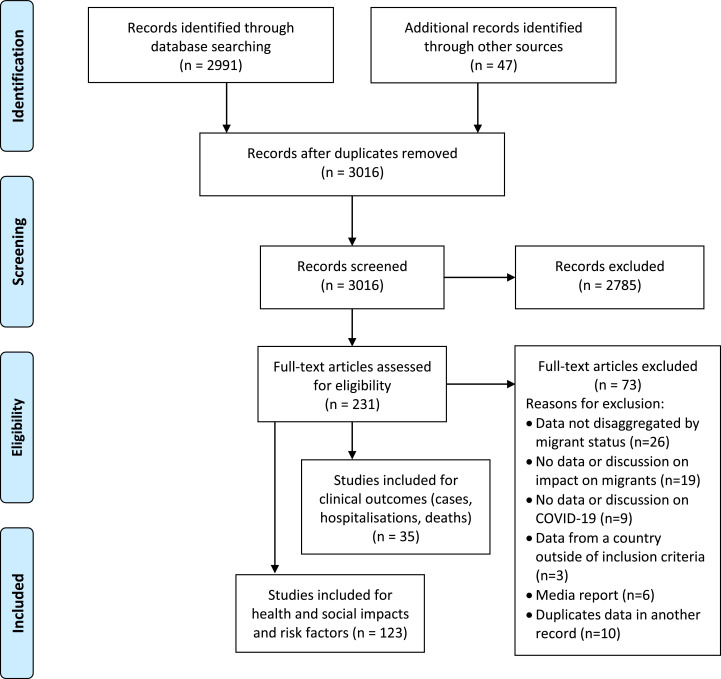
PRISMA flow diagram of included data sources.

Initial searches of databases and for grey literature identified 3016 records to screen; 158 of which were included in the final analysis (35 for primary outcomes, 123 for secondary outcomes) (Fig. 1). Supplementary Table 1 details the characteristics of all included data sources.

We found 35 data sources reporting on our primary clinical outcomes in migrants, including 21 on cases (Guttmann et al., 2020, Sundaram et al., 2020, Kim et al., 2020, Norweigan Institute of Public Health [Folkehelseinstituttet] 2020a, Norweigan Institute of Public Health [Folkehelseinstituttet] 2020b, Swedish Public Health Agency (Folkhälsomyndigheten) 2020, Statens Serum Institut 2020, Statens Serum Institut 2020, Guijarro et al., 2020, Grilli et al., 2020, Strully et al., 2020, Jaqueti Aroca et al., 2020, Chew et al., 2020, Alkhamis et al., 2020, Openshaw and Travassos, 2020, European Centre for Disease Prevention and Control 2020, Ministry of Health Singapore 2020, Ministry of Health Saudi Arabia 7 May 2020, Buda et al., 2020, Bozorgmehr et al., 2020), 4 on hospitalisations (Statens Serum Institut 2020, Giorgi Rossi et al., 2020, Hamadah and Behbehani, 2020, Fabiani et al., 2020), and 15 on mortality (Public Health England 2020, Statens Serum Institut 2020, Giorgi Rossi et al., 2020, Hamadah and Behbehani, 2020, Fabiani et al., 2020, Canevelli et al., 2020, Papon and Robert-Bobée, 2020, Observatoire Regional de Sante Ile de France 2020, Kunst et al., 2020, Hansson et al., 2020, Calderón-Larrañaga et al., 2020, Drefahl et al., 2020, Rostila et al., 2020, Centrum for epidemiologi och samhallsmediccin RS 2020, Cook et al., 2020). This includes data from Sweden (6 records), Italy (4), the United States (3), Canada (2), Denmark (2), Spain (2), the UK (2), France (2), Kuwait (2), Singapore (2), Norway (2), Germany (2), the Netherlands (1), Greece (1), Saudi Arabia (1) and across the EU/EEA/UK (1). Sources include peer-reviewed journal articles (13 records), pre-prints (3), national statistics (10), and other grey literature (9). A total of 59 studies were subjected to critical appraisal, including 22 primary outcomes and 37 secondary outcomes. Literature ranged in quality, with 28 studies fitting the criteria for high quality studies (80–100%), 19 for medium quality (60–79%) and 12 for low quality (0–59%). The average quality appraisal score was 73.6%, with reports included in the primary outcomes having a slightly higher quality score on average than those included in the secondary outcomes (74.9% and 72.9%, respectively). An additional 123 studies reported on indirect impacts of the pandemic on migrants and/or on risk factors for COVID-19 in migrants.

### 3.1 Clinical outcomes

Table 1 summarises included studies on the primary outcome (cases, hospitalisation, deaths).

**Table 1:** Data sources included in primary outcomes data, clinical outcomes (cases, hospitalisations, deaths).

Authors*	**Location**	**Population**	**Study design**	**Publication type**	**Study period**	**Sample size**	**Methods**	**Key results**	**Quality appraisal score (%)**
*COVID-19 cases*									
Guttman (Guttmann et al., 2020)	Ontario, Canada	Migrants and refugees	Population-based case/ testing data	Grey	To 13 June	624,386 tested	Rates of COVID-19 testing and percentage positivity in migrants, and relationship with socioeconomic factors	Migrants accounted for 43.5% of all COVID-19 cases but make up just over 25% of the population; migrants had lower rates of testing but a higher percentage positivity in those tested (refugees 10.4% positive, other migrants 7.6%, and Canadian-born 2.6%)	6/10 (60)
Sundaram (Sundaram et al., 2020)	Ontario, Canada	Migrants	Spatial comparison	Pre-print	1 March to 20 June	25,050 diagnoses	Association between COVID-19 diagnosis and percentage of migrants by area	Living in an area with a greater percentage of immigrants was positively associated with rate of COVID-19 diagnosis	8/8 (100)
Kim (Kim et al., 2020)	Washington DC, USA	Non-English speakers	Case/testing data	Peer-reviewed publication	29 February to 31 May	562,242 tested	Rates of COVID-19 testing and percentage positivity in non-English speakers versus English speakers	Non-English speakers were tested less frequently for COVID-19 (4.7% vs 5.6%) but were more likely to receive a positive result (18.6% vs 4.0%)	6/8 (75)
Norweigan Institute of Public Health (Norweigan Institute of Public Health [Folkehelseinstituttet] 2020a)	Norway	Migrants	Population-based case data	National statistics	To 4 May	7,847 cases	Percentage of reported cases that are among the foreign-born	Migrants made up 19% of reported cases in week 12 and 42% in week 18	N/A
Norweigan Institute of Public Health (Norweigan Institute of Public Health [Folkehelseinstituttet] 2020b)	Norway	Migrants	Population-based case data	National statistics	To 5 Nov	-	Incidence rate among migrants by country of origin versus those born in Norway	Until 1 July, incidence among the Somali-born was very high, but in autumn the risk increased for migrants from Pakistan, Iraq, Afghanistan, Serbia and Turkey	N/A
Swedish Public Health Agency (Swedish Public Health Agency (Folkhälsomyndigheten) 2020)	Sweden	Migrants	Population-based case data	National statistics	13 March to 7 May	-	Incidence of COVID-19 by country of birth	32% of cases were immigrants, despite making up 19% of the population; highest incidence among those born in Turkey, Ethiopia, and Somalia	N/A
Statens Serum Institut (Statens Serum Institut 2020)	Denmark	Migrants and their children	Population-based case data	National statistics	29 April to 6 May	-	Case, testing and incidence data, comparing migrants and their descendants with non-migrants	Non-Western migrants and their native-born children accounted for 18% of cases, despite making up 9% of the population	N/A
Statens Serum Institut (Statens Serum Institut 2020)	Denmark	Migrants and their children	Population-based data on cases, hospitalisation and mortality	National statistics	29 April to 7 September	-	Case, testing incidence, hospitalisation and mortality data, comparing migrants and their descendants with non-migrants	Non-Western migrants and their native-born children accounted for 26% of cases, and 15% of COVID-19 hospital admissions, despite making up 9% of the population	N/A
Guijarro (Guijarro et al., 2020)	Alcorcón, Spain	Migrants	Population-based cohort study	Pre-print	1 February to 25 April	152,018 residents	Incidence of COVID-19 in migrants versus Spaniards and relative risk by region of origin	Crude incidence of COVID-19 among migrants was higher than among Spaniards, at 8.71 and 6.51 per 1000 inhabitants respectively (p<0.001)	8/8 (100)
Grilli (Grilli et al., 2020)	Reggio Emilia, Italy	Migrants	Population-based case data	Peer-reviewed publication	6 March to 26 March	2635 tested	Odds of COVID-19 infection and being tested in migrants versus Italians	Immigrants and Italians had a similar prevalence of infection (OR 0.99, 95% CI 0.82-1.20) and similar probability of being tested (OR 0.93, 95% CI 0.81-1.10)	N/A
Strully (Strully et al., 2020)	USA	Migrants	Spatial comparison	Peer-reviewed publication	To 28 May	-	Association of proportion of migrants living in a region with COVID-19 case rates	Percentage of foreign-born residents was positively associated with COVID-19 case rate (fully adjusted IRR = 1.106, 95% CI 1.074-1.139, p<0.01) at county level	8/8 (100)
Jaqueti Aroca (Jaqueti Aroca et al., 2020)	Madrid, Spain	Migrants	Case/testing data	Peer-reviewed publication	To the second week of April	1,781 patients	Percentage positivity among foreign-born versus Spaniards in hospitals	No significant difference in percentage positivity between migrants and Spaniards (OR 1.08, 95% CI 0.95-1.24), but those from Latin America are at higher risk; only 12.5% of positive migrants were >65 years versus 56.9% Spaniards	8/10 (80)
Chew (Chew et al., 2020)	Singapore	Migrant workers	Case/testing data and clinical evaluation	Peer-reviewed publication	11 to 19 April	5,977 migrant workers	Review of data from an outbreak investigation among migrant workers in a dormitory, including test positivity and clinical parameters	1832 of 5977 migrant workers were symptomatic, of which 1264 (69%) were positive for COVID-19, corresponding to 21% of the cohort	6/10 (60)
Alkhamis (Alkhamis et al., 2020)	Kuwait	Migrant workers	Population-based case data	Peer-reviewed journal	23 February to 7 May	5988 cases	Modelling pandemic progression (spatiotemporal cluster analysis) in Kuwait citizens/ residents and migrant workers	78.8% of COVID-19 cases were in migrant workers, 40.1% of which were of Indian nationality; significant spreading events among migrant workers	6/10 (60)
Openshaw (Openshaw and Travassos, 2020)	USA	Migrants in detention centres	Viewpoint (in	Peer-reviewed publication	To May	-	Reports COVID-19 cases at ICE detention centres	Over 1200 confirmed COVID-19 cases across 52 facilities run by ICE	6/6 (100)
ECDC (European Centre for Disease Prevention and Control 2020)	EU/EEA and UK	Migrants in detention centres	Report reporting cases	Grey	To June	-	Reports COVID-19 cases at detention centres in Europe	Reported outbreaks in detention centres in Germany and Portugal	6/6 (100)
Ministry of Health (Ministry of Health Singapore 2020)	Singapore	Migrant workers	Population-based case data	National statistics	18 Nov	5,704,000 (population)	Surveillance of new confirmed cases in the past 14 days, including proportion in dorm residents	54,502 (95.7%) of 58,135 all in-country cases of COVID-19 were in migrants residing in dormitories	2/10 (20)
Ministry of Health (Ministry of Health Saudi Arabia 7 May 2020)	Saudi Arabia	Migrants	Population-based case data	National statistics	To 7 May	-	Routine surveillance	75% of all people in-country who have tested positive for COVID-19 were migrants	N/A
Greek Ministry of Health (Greek Ministry of Health 2020)	Greece	Migrants and refugees	Hospital-based case data	National statistics	To 16 September	-	Routine surveillance	Almost half of COVID-19 patients hospitalised in Attica are refugees from camps/hosting sites or destitute migrants	N/A
Buda (Buda et al., 2020)	Germany	Refugees	Outbreaks case data	National statistics	To 11 August	-	Collation of outbreak reports, assessing the proportion in refugee centres (vs other settings such as nursing homes)	2.5% of notified outbreaks (199 of 7864) were reported in refugee centres, comprising 7.5% (n=4,146) of all notified cases during outbreaks (n=55,141)	N/A
Bozorgmehr (Bozorgmehr et al., 2020)	Germany	Refugees in reception/ accommodation centres	Outbreaks case data	Grey	To 22 May	9785 refugees	Meta-analysis of media reports in Germany to identifypooled cumulative incidence rate in refugee reception/ accommodation centres	Identified 42 outbreaks in 11 federal states, with 1769 confirmed cases; IR of 17.0% (95% CI 12.0 to 23.0, I^2^ = 98.3%)	N/A
*COVID-19 hospitalisation*									
Giorgi Rossi (Giorgi Rossi et al., 2020)	Reggio Emilia, Italy	Migrants	Population-based cohort study	Peer-reviewed publication	27 February to 2 April	2,653 tested	COVID-19 incidence, hospitalisation and death in migrants versus Italians	Immigrants had a higher risk of hospitalisation (HR 1.3, 95% CI 0.99-1.81) than Italians	7/10 (70)
Hamadah (Hamadah H and Behbehani, 2020)	Kuwait	Migrants	Hospital-based cohort study	Peer-reviewed publication	24 February to 20 April	1,123 patients	Comparison of ICU admission, ARDS, pneumonia and mortality in migrants and non-migrants	Migrants had increased odds of death or ICU admission (OR 2.14, 95% CI 1.12-4.32), ARDS (OR 2.44, 95%CI 1.23-5.09) and pneumonia (OR 2.24, 95% CI 1.27-4.12)	8/8 (100)
Fabiani (Fabiani et al., 2020)	Italy	Migrants	Population-based clinical and mortality data	Pre-print	20 Feb to 19 July	213,180 cases	Comparison of case fatality rate and rate of admission to hospital and ICU between migrants versus Italians	Non-Italian cases were diagnosed at a later date than Italian cases and were more likely to be hospitalised (ARR=1.39, 95% CI 1.33- 1.44) and admitted to an ICU (ARR=1.19, 95% CI 1.07-1.32)	8/8 (100)
*COVID-19 mortality*									
Canevelli (Canevelli et al., 2020)	Italy	Migrants	Temporal comparison	Peer-reviewed publication	21 February to 29 April	2,687 deceased cases	Comparison of proportion of migrants in COVID-19 deaths versus all-cause mortality in 2018	The proportion of migrants and non-migrants among COVID-related deaths (2.5% and 97.5% respectively) was similar to the estimated 2018 all-cause mortality rates (2.6% and 97.4%); but migrants were younger at the time of death versus non-migrants (71.1, SD 13.1 vs 78.3, SD 10.8, p<0.001)	9/10 (90)
Public Health England (Public Health England 2020)	England	Migrants	Temporal comparison	Grey	21 March to 8 May	-	Comparison of all-cause mortality in 2020 versus 2014-2018 in migrants and UK-born	Deaths in 2020 were over 3 times higher than 2014-2018 for those from Central and Western Africa, the Caribbean, South East Asia, Middle East, and South and Eastern Africa, versus 1.7 times higher overall in England	8/8 (100)
Papon (Papon and Robert-Bobée, 2020)	France	Migrants	Temporal comparison	Grey	March to April	-	Comparison of proportion of migrants in registered deaths in 2020 versus 2014-2019	The foreign-born represented 15% of registered deaths in March and April 2020 versus 13% in March and April 2019	6/10 (60)
Observatoire Regional de Sante Ile de France (Observatoire Regional de Sante Ile de France 2020)	Paris, France	Migrants	Spatial comparison	Grey	March 2020	Not stated	Mortality (daily deaths) by Parisian departments (areas) compared with sociodemographic characteristics of the department.	Eg. Seine-Saint-Denis, a district in the north of Paris where 30% of the population is an immigrant, had a 188% mortality increase compared with 2019 versus a 96% increase in Paris as a whole	1/10 (10)
Kunst (Kunst et al., 2020)	Netherlands	Migrants and their children	Temporal comparison	National statistics	March to April	-	Comparison of mortality in March-April versus in the preceding weeks, adjusted for seasonal factors, in migrants versus Dutch	Mortality was 47% higher than expected for immigrants from non-Western countries and their children, 49% higher for immigrants from Western countries and their children, and 38% higher for the native-born with Dutch parents	N/A
Hansson (Hansson et al., 2020)	Sweden	Migrants	Temporal comparison	Peer-reviewed publication	February to May	-	Comparison of all-cause mortality in 2020 versus 2016-2019 by region of birth	Among middle-aged (40-64 years) and older (>65 years) people born in Syria, Iraq and Somalia excess mortality was ~220%; among those born in Sweden, the EU, the Nordic countries or North America, excess mortality among those >65 was 19% and among the middle aged was 1%	N/A
Calderón-Larrañaga (Calderón-Larrañaga et al., 2020)	Stockholm, Sweden	Migrants	Spatial/ temporal comparison	Peer-reviewed publication	6-12 April	2,379,792 residents	Comparison of excess mortality compared with previous 5 years in areas according to share of migrants	Areas with the lowest tercile share of Swedish-born had 178% excess mortality compared with the previous 5 years	6/10 (60)
Drefahl (Drefahl et al., 2020)	Sweden	Migrants	Individual-level survival analysis	Peer-reviewed publication	To 7 May	1,189,484 py (17,181 deaths)	Risk of death from COVID-19 in individual-level data according to migrant status and region of origin	Immigrants from LMICs from the Middle East and North Africa showed increased mortality among men (HR 3.13, 95% CI 2.51-3.90) and women (HR 2.09, 95% CI 1.52-2.89) as compared to the Swedish-born	8/10 (80)
Rostila (Rostila et al., 2020)	Stockholm, Sweden	Migrants	Population-based cohort study	Grey	31 Jan to 4 May	1,778,670 individuals	Risk of death from COVID-19 in individual-level data according to migrant status and region of origin	Migrants from Middle Eastern countries (RR 3.2, 95% CI 2.6-3.8), Africa (RR 3.0, 95% CI 2.2-4.3) and the Nordic countries (RR 1.5, 95% CI 1.2-1.8) had higher COVID-19 mortality versus the Swedish-born	9/11 (82)
Centrum for epidemiologi och samhallsmediccin (Centrum for epidemiologi och samhallsmediccin RS 2020)	Stockholm, Sweden	Migrants	Individual-level survival analysis	Grey	To 30 June	-	Risk of death from COVID-19 in individual-level data according to country of birth, among those aged 25 years and older	Migrants from Somalia (HR 12.39, 95% CI 7.93-19.36), Lebanon (HR 6.19, 95% CI 3.41-11.24), and Syria (HR 6.14, 95% CI 4.28-8.80) show increased risk of death compared with Swedish-born, adjusted for age and sex	N/A
Cook (Cook et al., 2020)	UK	Migrant healthcare workers (HCWs)	Characterisation of reported HCW deaths	Grey	To 22 April	106 HCWs	Proportion of UK healthcare workers who died who were born outside the UK	Of 106 UK healthcare workers who died up until 22 April 2020, at least 56 (53%) were born outside the UK	4/10 (40)

*Where papers report on multiple outcomes (cases, hospitalisations, deaths) papers are included under the first relevant sub-heading only

#### 3.1.1 COVID-19 cases

Data that disaggregate COVID-19 incidence and testing uptake by migrant status indicate that migrants account for a disproportionate number of COVID-19 infections despite low rates of testing. In Ontario, Canada, immigrants make up just over 25% of the population, but accounted for 43.5% of all COVID-19 cases up until 13 June (Guttmann et al., 2020). Immigrants had lower rates of testing but there was a higher percentage of positive cases in those tested. Refugees had the highest percentage positivity, at 10.4%, compared with 7.6% among other immigrants, and 2.6% in the Canadian-born. Migrants and refugees from Central, Western and East Africa, South America, the Caribbean, Southeast Asia and South Asia showed the highest rates of positive cases for COVID-19 (Guttmann et al., 2020). Among all women who tested positive, 36% were employed as healthcare workers (immigrants and refugees made up 45% of these positive healthcare workers): 55% of positive cases were among female migrants in the economic caregiver categories, including 53% among those from the Philippines, 64% from Jamaica and 76% from Nigeria (Guttmann et al., 2020). In another study, living in an area of Ontario with a greater percentage of recently arrived migrants was significantly positively associated with an increased rate of COVID-19 diagnoses (Sundaram et al., 2020).

In the US, a study that reports using language as a surrogate for immigration status (in the absence of routine data collection on migrant status) found that non-English speakers were tested less frequently for COVID-19 (29 February to 31 May) (4.7% [95% CI 4.5%–4.9%] vs 5.6% [95% CI 5.6%–5.7%]), with variations across language groups, but were more likely to test positive (18.6% [95% CI 16.8%–20.4%] vs 4.0% [95% CI 3.8%–4.2%]) (Kim et al., 2020). Fewer years of formal education and a lack of English or French language ability at the time of immigration was associated with lower testing rates and higher percentage positivity among recent adult migrants in Ontario, Canada (Guttmann et al., 2020).

In Norway, migrants made up 19% of all reported cases in the week starting 16 March, rising to 42% in the week starting 27 April (Norweigan Institute of Public Health [Folkehelseinstituttet] 2020a). While incidence among the Somali-born was very high until 1 July, in the autumn the risk increased for migrants from Pakistan, Iraq, Afghanistan, Serbia and Turkey (Norweigan Institute of Public Health [Folkehelseinstituttet] 2020b). Similarly in Sweden, during the first peak of the pandemic (13 March to 7 May), 32% of positive cases were immigrants, despite making up only 19% of the population (Swedish Public Health Agency (Folkhälsomyndigheten) 2020). The incidence of COVID-19 was highest among migrants from Turkey (753 per 100,000), followed by Ethiopia (742 per 100,000) and Somalia (660 per 100,000). This compares with an incidence of 189 per 100,000 for non-migrants who were born in Sweden for the same time-period (Swedish Public Health Agency (Folkhälsomyndigheten) 2020).

In Denmark, non-Western migrants and their native-born children accounted for 18% of cases (29 April to 6 May), which was double their share in the Danish population (Statens Serum Institut, 2020). In an later update (7 September), this had risen substantially to migrants accounting for 26% of cases Statens Serum Institut (2020). Among non-Western migrants, the COVID-19 incidence rate was 315 per 100,000 compared with 240 per 100,000 for non-Western descendants and 128 per 100,000 among ethnic Danes (29 April to 6 May) (Statens Serum Institut, 2020). Particularly high incidence rates were seen among migrants from Morocco, Pakistan, Somalia and Turkey (Statens Serum Institut 2020, Statens Serum Institut 2020).

In Alcorcón, Spain (to 25 April) the crude incidence rate of COVID-19 among migrants was higher than among the host Spanish populations, at 8.71 and 6.51 per 1000 inhabitants respectively (*p* < 0.001) (Guijarro et al., 2020). The relative risk for COVID-19 was elevated in migrants from sub-Saharan Africa (RR 3.66, 95% CI 1.42–9.41; *p* = 0.007), the Caribbean (RR 6.35, 95% CI 3.83–10.55; *p* < 0.001), and Latin America (RR 6.92, 95% CI 4.49–10.67; *p* < 0.001) but not from other regions (Guijarro et al., 2020). Data from a hospital in Madrid up to the second week of April showed no significant differences between migrants and host population in terms of COVID-19 positivity among those tested (52.5% [136/259] vs 51.4% [782/1522]). There was also no difference in testing rate (odds ratio [OR] 1.08 95% CI 0.95-1.24) between migrants and the host population; only 12.5% of COVID-19 positive migrants were older than 65 years of age, compared to 56.9% of Spanish citizens who tested positive. Migrants from Latin America had higher positivity rates per 1000 people, compared with the host population and other migrant groups (Jaqueti Aroca et al., 2020).

A US study found that being foreign-born was positively associated with COVID-19 case rate at the county level (data to 28 May; with fully adjusted incidence rate ratio 1.106, 95% CI 1.074–1.139; *p* < 0.01) (Strully et al., 2020).

In Singapore, labour migrants in crowded dormitories have been disproportionately impacted by COVID-19, with over 95% of confirmed cases (to 19 June) among dormitory-housed migrants; as of 18 Nov, 54,502 (95.7%) of 58,135 all in-country cases of COVID-19 were in migrants residing in dormitories (Ministry of Health Singapore 2020). A study in one isolated dormitory of 5977 migrant workers (mean age 33 years) in an accommodation centre of 13,000 migrants, 1264 tested positive for COVID-19 (between 11 to 19 April) (Chew et al., 2020). Similarly in Saudi Arabia, Ministry of Health Data reported that 75% of all people in-country who had tested positive for COVID-19 were migrants (to 7 May) (Ministry of Health Saudi Arabia, 2020).

Data on migrants in detention facilities and reception centres suggest these are high-risk settings for COVID-19. In the US, across 52 facilities run by the Department of Homeland Security (DHS)’s Immigration and Customs Enforcement (ICE) agency as of May 2020 more than 50% of ICE migrant detainees who had been tested were positive (Openshaw and Travassos, 2020). The European Centre for Disease Prevention and Control has also highlighted several examples of COVID-19 outbreaks in migrant reception and detention centres in the European Union/European Economic Area (EU/EEA) and the United Kingdom (UK) in a technical report, including Greece, Germany, Malta, The Netherlands, and Portugal, and concludes that whilst there is no evidence to suggest that SARS-CoV-2 transmission is higher amongst migrants and refugees, overcrowding in reception and detention centres may increase their exposure to the disease (European Centre for Disease Prevention and Control 2020).

This is in line with national notification data from Germany where 2.5% of notified outbreaks up to 11 August (199 of a total of 7864) were reported in refugee centres comprising 7.5% (*n* = 4,146) of all notified cases during outbreaks (*n* = 55,141) across the country. The average number of cases per outbreak in refugee centres was 20.8, higher than in any other outbreak setting (Buda et al., 2020). A systematic analysis of outbreak reports to 22 May identified 42 outbreaks in refugee reception and district accommodation centres of 11 federal states, with 1781 confirmed SARS-CoV2 cases among 9785 refugees in those centres. The pooled cumulative incidence rate (attack rate) was reported as 17.0% (95% CI 12.0–23.0, I2 = 98.3%) (Bozorgmehr et al., 2020).

A temporal and spatiotemporal dynamics study of the COVID-19 pandemic in Kuwait using daily confirmed case data collected between the 23 February and 7 May concluded that densely populated areas and poor living conditions of migrant workers resulted in the highest number of significant spreading and clustering events within their communities (Alkhamis and Khajah, 2020).

We found one Italian study reporting no differences between migrants and non-migrants in terms of the probability of being tested (OR 0.93; 95% CI 0.81–1.1) and a similar prevalence of infection (OR 0.99; 95% CI 0.82–1.20) (Grilli et al., 2020).

#### 3.1.2 Hospitalisation due to COVID-19

In a prospective COVID-19 registry study (*n* = 1123) comparing Kuwaitis with non-Kuwaitis/migrants (two-thirds of the Kuwaiti population are migrants, the majority of non-Kuwaitis are migrant workers) in the main COVID-19-specific healthcare facility in the country, with adjustments made to age, gender, smoking and selected co-morbidities, non-Kuwaitis (91.6% males; mean age 41.0 years) had two-fold increase in the odds of death or being admitted to the intensive care unit compared to Kuwaitis (OR 2.14, 95% CI 1.12–4.32). Non-Kuwaitis also had higher odds of acute respiratory distress syndrome [ARDS] (OR 2.44, 95% CI 1.23–5.09) and pneumonia (OR 2.24, 95% CI 1.27–4.12) (Hamadah and Behbehani, 2020).

In Denmark, non-Western migrants and their children accounted for 15% of COVID-19 hospital admissions (to 7 September), despite only making up 9% of the population Statens Serum Institut (2020).

In one province in Italy (27 February to 2 April), migrants were found to have a higher risk of hospitalisation (hazard ratio [HR] 1.3, 95% CI 0.99–1.81) than Italians (Giorgi Rossi et al., 2020). In Italian surveillance data (to 19 July) non-Italian cases were diagnosed at a later date than Italian cases and were more likely to be hospitalised (adjusted relative risk 1.39 [95% CI 1.33–1.44]) and admitted to an intensive care unit (1.19 [95% CI 1.07–1.32]), especially in those coming from lower human development index countries (Fabiani et al., 2020).

In Greece, almost half of COVID-19 patients hospitalised in Attica (Athens and surrounding areas) as of 17 Sept were refugees from camps/hosting sites and destitute migrants from the city centre, including in Sotiria hospital (40 of 103 are refugees), Evaggelismos (36 of 66), Amalia Fleming (10 of 20) and Attikon (26 of 26); many of these patients were reported to be “asymptomatic and young” but could not be returned to overcrowded accommodation (Greek Ministry of Health 2020).

#### 3.1.3 COVID-19 mortality and excess deaths

An analysis of all recorded COVID-19 deaths (to 7 May) in Sweden found that being an migrant from an LMIC is predictive of a higher risk of death from COVID-19, but not for all other causes of death (Drefahl et al., 2020). In models adjusting for age and sociodemographics, migrants from LMICs from the Middle East and North Africa had a three times higher mortality rate from COVID-19 among men (HR 3.13, 95% CI 2.51–3.90) and two times higher mortality among women (HR 2.09, 95% CI 1.52–2.89) as compared to the Swedish-born (Drefahl et al., 2020). Similarly, data from Stockholm, Sweden until 4 May shows that migrants from Middle Eastern countries (RR 3.2, 95% CI 2.6–3.8), Africa (RR 3.0, 95% CI 2.2–4.3) and the Nordic countries (RR 1.5, 95% CI 1.2–1.8) had higher COVID-19 mortality when compared to Swedish-born people, adjusting for age, sex and sociodemographic characteristics. Especially high mortality risks from COVID-19 were found among individuals born in Somalia (RR 8.9, 95% CI 5.6-14.0), Lebanon (RR 5.9, 95% CI 3.4–10.3) and Syria (RR 4.7, 95% CI 3.3–6.6) (Rostila et al., 2020).

An epidemiological report that compared risk of death from COVID-19 in over 25-year olds who were foreign-born versus Swedish-born of the same age to 30 June in Stockholm Country found marked differences between Swedish-born and Somali (HR adjusted for age and sex 12.39 [7.93–19.36]), Lebanese (6.19 [3.41–11.24]), and Syrian (6.14 [4.28–8.80]) migrants (Hansson et al., 2020). These effects were attenuated when adjusted for neighbourhood, education level, occupation, income, household size and previous chronic illness, but remained higher among migrants than Swedish-born (Centrum for epidemiologi och samhallsmediccin RS 2020). In a brief report of 106 healthcare workers who died in the UK up until 22 April 2020, 56 (53%) were reportedly born outside the UK (Cook et al., 2020).

No differences in mortality from COVID-19 by migration status were observed in crude analyses by migrant status in Denmark (data to 7 September) (Statens Serum Institut 2020). In one province of Italy, migrants were found to have a similar risk of death to non-migrants (27 February to 2 April) (Giorgi Rossi et al., 2020). However, Italian surveillance data from the start of the outbreak to 19 July found an increased risk of death in non-Italians from low-Human Development Index countries (adjusted RR 1.32, 95% CI 1.01-1.75) (Fabiani et al., 2020).

Definitional and data collection challenges mean that attention has focused on all cause excess mortality during the pandemic, comparing deaths with those expected on the basis of rates in preceding years. In England, for example (21 March to 8 May) where the number of death registrations from all causes was 1.7 times higher than the average during the same period in 2014-2018, the relative increase in total deaths was greater among those born outside the UK; deaths in 2020 were over 3 times higher than the equivalent period in 2014 to 2018 for those from Central and Western Africa (4.5 times higher) the Caribbean (3.5), South East Asia (3.4), Middle East (3.2) and South and Eastern Africa (3.1). For migrants born in other countries within the EU (internal migrants) the level of increased risk was similar to those born in the UK (Public Health England, 2020).

In France, foreign-born people represented 15% of registered deaths (March and April 2020) versus 13% for the same period in 2019. This includes an increase of 54% deaths among migrants from North Africa (Algeria, Morocco, Tunisia), 114% for those from sub-Saharan Africa, and 91% for those from Asia. Migrants from other parts of Europe, America or Oceania had similar mortality rates to the French-born, who experienced a 22% excess mortality (Papon and Robert-Bobée, 2020). This same trend is also seen in different regions of France; for example Seine-Saint-Denis, a district in the north of Paris where 30% of the population are immigrants, saw a 188% mortality increase compared with 2019, versus a 96% increase in Paris as a whole (Observatoire Regional de Sante Ile de France 2020).

In the Netherlands (9 March to 19 April 2020), mortality was 47% higher than expected for migrants from non-Western countries and their immediate children (based on number of deaths in the preceding weeks, adjusted for seasonal factors), 49% higher for migrants from Western countries and their children, and 38% higher for the native-born people with Dutch parents (Kunst et al., 2020).

In Sweden, mortality among migrants was elevated in 2020 compared with previous years. A comparison between all-cause mortality data from March to May 2020 with data from the same period in 2016 to 2019 found that among middle-aged (40–64 years) and older (>65 years) migrants born in Syria, Iraq and Somalia excess mortality was approximately 220%. Among people born in Sweden, the EU, the Nordic countries or North America, the excess mortality among those >65 was 19% and among the middle aged was 1% (Hansson et al., 2020). In Stockholm during the peak of the epidemic (6 to 12 April 2020), areas with the lowest tercile of share of Swedish-born had 178% excess mortality compared with the previous five years (Calderón-Larrañaga et al., 2020).

In Italy, on the other hand, between 21 February and 29 April 2020, found the share of migrants and non-migrants among COVID-related deaths (2.5% and 97.5% respectively) was similar to their share in all-cause mortality rates estimated in Italy in 2018 (2.6% and 97.4% respectively) (Canevelli et al., 2020). However, migrants were younger at the time of death than non-migrants (71.1, standard deviation [SD] 13.1 years vs 78.3, SD 10.8 years, *p* < 0.001).

### 3.2 Indirect health and social impacts

The mental health impact of the COVID-19 pandemic and associated restrictions has been well-documented. Migrants may be particularly affected due to pre-existing risk factors (Júnior et al., 2020, Falicov et al., 2020) and potential exclusion and social isolation (Doctors of the World 2020), and worsening of pre-existing mental health conditions (Pinzón-Espinosa et al., 2020, Endale et al., 2020); providing remote therapy for these individuals can be challenging (Mattar and Piwowarczyk, 2020). In one Canadian study, however, immigrants were found to be less likely to increase negative health behaviours than Canada-born adults (Zajacova et al., 2020). In a nationally representative US survey carried out in March 2020, COVID-19-related fear and associated anxiety and depressive symptoms were higher for migrants compared with the US-born (*p* < 0.001) (Fitzpatrick et al., 2020), with similar findings in other studies (Goodman et al., 2020, Choi et al., 2020). In a cross-sectional survey of 295 Filipino domestic helpers in Hong Kong, multivariate regression results showed that the insufficiency of personal protective equipment (PPE) (OR=1.58 [95% CI 1.18–2.11]), increased workload (OR 1.51 [95% CI 10.2–2.25]), and concerns about being forced out of their jobs if they test positive for COVID-19 (OR 1.32 [95% CI 1.04–1.68]) were significantly associated with anxiety in a multivariate analysis (Yeung et al., 2020).

Migrants may be especially impacted by travel restrictions (Guadagno, 2020, Migration Data Portal 2020, Zajacova et al., 2020). Arriving migrants have been pushed back or quarantined at borders and forced to stay in informal or overcrowded transit sites, while international refugee resettlement programmes have been disrupted (Guadagno, 2020, Jauhiainen, 2020). For migrants who are already settled, but not considered resident, border restrictions may force them to overstay their visas, or prevent them from visiting family or friends outside of their host country, exacerbating feelings of isolation (Kanlungan Filipino Consortium 2020). Concerns have also been raised that border closures may increase smuggling of migrants (Sarrica et al., 2020). COVID-19 may meanwhile pose a barrier to integration for migrants and refugees (Falkenhain et al., 2020), for example due to the suspension and modification of resettlement schemes (Brickhill-Atkinson and Hauck, 2020, Rush, 2020), and education programmes (Brickhill-Atkinson and Hauck, 2020, Primdahl et al., 2020, Mupenzi et al., 2020, OECD 2020, Silverman et al., 2020). Migrants who are particularly vulnerable may be disproportionality affected by the negative social impact of lockdown (Zero and Geary, 2020, Sabri et al., 2020). Migrants are considered to be especially vulnerable to job loss and economic hardship as a result of COVID-19 (Kanlungan Filipino Consortium 2020, Brickhill-Atkinson and Hauck, 2020, OECD 2020, Wang et al., 2020, Dempster and Zimmer, 2020, Basso et al., 2020, Davis, 2020, Borjas and Cassidy, 2020, Pacheco et al., 2020, Garrote Sanchez et al., 2020). A qualitative cumulative risk assessment for migrant workers in Kuwait found many workers are now facing layoffs, furloughs, non-payment and late payment of wages putting them in significant financial hardship (Alahmad et al., 2020). Across Organisation for Economic Cooperation and Development (OECD) countries, approximately 30% of migrants are considered to be living in relative poverty, compared with 20% of the native-born people (OECD, 2020), which increases their vulnerability to COVID-19 infection (Doctors of the World 2020, Valeriani et al., 2020).

Migrants may also be experiencing discrimination as a result of the COVID-19 pandemic (Guadagno, 2020, Patel et al., 2020, Centre for British-Turkish Understanding 2020). In particular, Chinese and other Asian migrants have been targeted due to the original emergence of the pandemic in China, with reports of bullying, awkward behaviour, avoidance of Chinese restaurants and shops, and physical attacks (Bofulin, 2020, Cross and Benson, 2020, Aydemir and Akyol, 2020). In surveys and interviews with people of Chinese origin living in France, nearly a third reported having experienced at least one discriminatory act since January 2020 (Wang et al., 2020).

### 3.3 Risk factors and vulnerabilities for COVID-19 in migrants

**Fig. 2:**
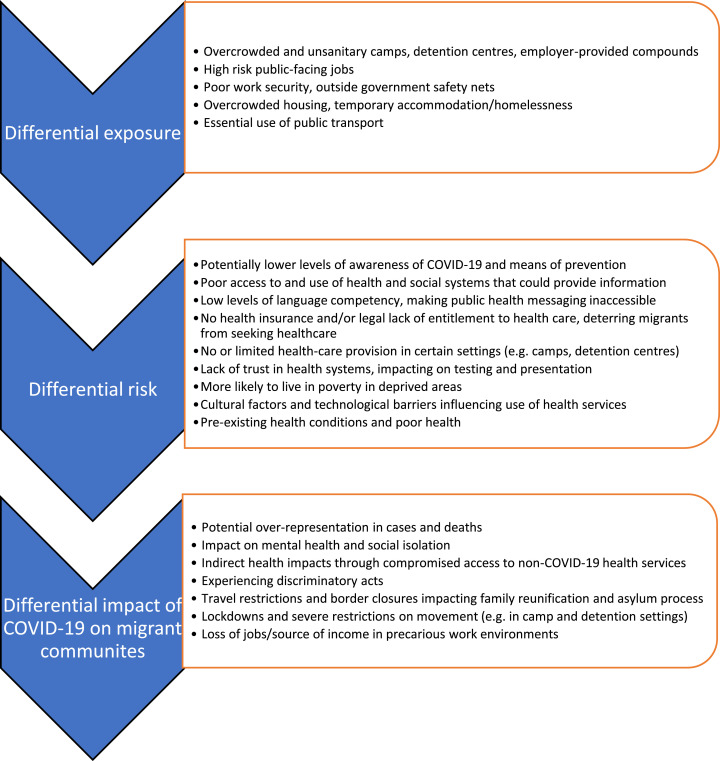
Migrant-specific risk factors and vulnerabilities identified in included literature.

Table 2 summarises key risk factors for migrants for COVID-19 reported from included data sources. Fig. 2 highlights key risk factors and vulnerabilities of migrants identified in the literature.

**Table 2:** Risk factors and vulnerabilities reported for migrants for COVID-19.

**Authors**	**Location**	**Population**	**Risk factors**				
			**Co-morbidities**	**Health seeking and health care**	**Social and cultural**	**Occupation**	**Details**
Migration Data Portal (Migration Data Portal 2020)	Global	Migrants				x	Occupational risk (frontline/essential, HCWs)
Júnior (Júnior et al., 2020)	Global	Refugees		x	x		Conditions in camps (overcrowding, sanitation, healthcare, language/culture)
DotW (Doctors of the World 2020)	England	Refugees, asylum seekers, undocumented migrants	x	x	x	x	Socio-economic determinants; barriers to healthcare
Valeriani (Valeriani et al., 2020)	Sweden	Migrants	x	x	x	x	Socio-economic determinants; occupational risk (frontline); barriers to healthcare
Brickhill-Atkinson (Brickhill-Atkinson and Hauck, 2020)	Global	Refugees	x	x	x	x	Overcrowding; comorbidities; occupational risk (frontline); barriers to healthcare (language, technological)
Page (Page et al., 2020)	USA	Undocumented migrants		x			Barriers to healthcare (immigration status, cultural, language)
Langellier (Langellier, 2020)	USA	Non-citizens		x	x	x	Socio-economic determinants; occupational risk (frontline); barriers to healthcare
Clarke (Clarke et al., 2020)	USA and Canada	Refugee	x	x	x	x	Socio-economic determinants; occupational risk; barriers to healthcare; co-morbidities
Wang (Wang et al., 2020)	Global	Migrant workers		x	x	x	Socio-economic determinants; occupational risk; barriers to healthcare
Kanlungan Filipino Consortium (Kanlungan Filipino Consortium 2020)	UK	Filipino precarious migrants		x	x	x	Occupational risk (frontline, job security); barriers to healthcare; overcrowding
Capps (Capps R, Gelatt, 2020)	USA	Migrant workers		x			Barriers to healthcare (immigration status)
Zelaya (Zelaya et al. 2020)	USA	Undocumented migrants		x			Barriers to healthcare (immigration status)
Davis (Davis, 2020)	Massachusetts, US	Migrant households		x			Barriers to healthcare (immigration status; language/cultural)
Patel (Patel et al., 2020)	UK	Migrants and refugees		x	x		Barriers to healthcare (immigration status; language/cultural); conditions in detention centres (overcrowding)
Cross (Cross and Benson, 2020)	US	Undocumented migrants		x	x	x	Barriers to healthcare (immigration status); occupational risk (frontline); conditions in detention centres
Gosselin (Gosselin et al., 2020)	France	Migrants	x	x	x	x	Co-morbidities; barriers to healthcare (language/cultural); occupational risk (frontline); conditions in camps and detention centres
Elisabeth (Elisabeth et al., 2020)	Sweden	Refugees	x	x	x		Co-morbidities; socio-economic determinants (poverty, overcrowding); barriers to healthcare (language/cultural)
Tadolini (Tadolini et al., 2020)	Global	Migrants	x				Co-infection with tuberculosis
Motta (Motta et al., 2020)	Global	Migrants	x				Co-infection with tuberculosis
Dias (Dias et al., 2020)	Portugal	Migrants		x	x	x	Living conditions; occupational risk; barriers to healthcare
Iacobucci (Iacobucci, 2020)	Greece	Refugees in camps	x	x	x		Conditions in camps (overcrowding, healthcare); co-morbidities
Hargreaves (Hargreaves et al., 2020)	Global	Migrants and refugees	x	x	x	x	Conditions in camps (overcrowding, sanitation, healthcare); occupational risk (living conditions); co-morbidities
Jozaghi (Jozaghi and Dahya, 2020)	Global, with focus on Canada	Refugees in camps	x		x		Conditions in camps (overcrowding, sanitation); co-morbidities
ACAPS (ACAPS 2020)	Greece	Refugees in camps			x		Conditions in camps (overcrowding, sanitation, healthcare)
Wood (Wood and Devakumar, 2020)	England	Migrant children		x			Barriers to healthcare (immigration status)
Germain (Germain and Yong, 2020)	England	Migrant women		x			Barriers to healthcare (immigration status)
Cholera (Cholera et al., 2020)	USA	Migrant children		x	x		Barriers to healthcare (immigration status); multigenerational households
Bakhiet (Bakhiet et al. 2020)	USA	Refugees		x			Barriers to healthcare (immigration status, other structural/cultural)
Greenaway (Greenaway et al., 2020)	Global	Migrants	x	x	x	x	Socio-economic determinants; barriers to healthcare
Wilson (Wilson et al., 2020)	USA	Undocumented migrants		x			Barriers to healthcare (immigration status)
Behbahani (Behbahani et al., 2020)	New York, USA	Migrants		x			Barriers to healthcare (immigration status, language)
Lopez (Lopez and Holmes, 2020)	USA	Migrants		x			Barriers to healthcare (immigration status)
Lam (Lam, 2020)	Canada	Migrant sex workers		x		x	Barriers to healthcare (immigration status); occupational risk
Doyle (Doyle, 2020)	Canada	Migrant workers		x	x	x	Barriers to healthcare (immigration status); occupational risk; overcrowding
Bodenmann (Bodenmann et al., 2020)	Vaud, Switzerland	Forced migrants		x	x		Barriers to healthcare (cultural, language); socio-cultural factors; overcrowding
Institut For Menneske Rettigheder (Institut for Menneskerettigheder 2020)	Denmark	Migrants		x	x		Language and cultural barriers to communication of govt guidance
Ceccarelli (Ceccarelli et al., 2020)	Rocca di Papa, Italy	Migrants in reception centre		x	x		Low awareness of pandemic
Guo (Guo et al., 2020)	Spain	Chinese migrants		x	x		High awareness of pandemic and compliance
Zhang (Zhang and Zhao, 2020)	Global	Chinese migrants		x	x		High awareness of pandemic and compliance
Vonen (Vonen et al., 2020)	Europe	Refugees in camps		x	x		Conditions in camps (overcrowding, sanitation, healthcare)
Medact (Medact 2020)	Europe	Refugees in camps		x	x		Conditions in camps (testing/ healthcare)
Hernandez Suarez (Hernandez-Suarez et al., 2020)	Global	Refugees in camps		x			Potential for transmission in camps; healthcare impact
Hargreaves (Hargreaves et al., 2020)	Europe	Refugees in camps and detention centres		x	x		Conditions in camps and detention centres (overcrowding, sanitation, healthcare)
Alawa (Alawa et al., 2020)	Global	Refugees in camps		x	x		Conditions in camps (overcrowding, sanitation, healthcare)
Peprah (Peprah, 2020)	Global	Older refugees in camps		x	x		Conditions in camps (sanitation, healthcare, trauma)
Spernovasilis (Spernovasilis et al., 2020)	Greece	Refugees in camps		x	x		Conditions in camps (overcrowding, sanitation, healthcare)
Kondilis (Kondilis et al., 2020)	Greece	Refugees in camps		x	x		Conditions in camps (overcrowding, sanitation, healthcare)
The Lancet (The Lancet 2020)	Global	Refugees in camps		x	x		Conditions in camps (overcrowding, sanitation, healthcare)
Gilman (Gilman et al., 2020)	Moira, Greece	Refugees in camps		x	x		Potential for transmission in camps (due to overcrowding, sanitation, healthcare)
Alemi (Alemi et al., 2020)	Global	Refugees in camps	x	x	x		Conditions in camps (overcrowding, sanitation, stigma deterring health seeking); comorbidities
Logar (Logar and Leese, 2020)	Italy	Child migrants in detention centres	x		x		Conditions in detention centres (overcrowding); comorbidities
Meyer (Meyer et al., 2020)	USA	Migrants in detention centres		x	x		Conditions in detention centres (overcrowding, healthcare)
Irvine (Irvine et al., 2020)	USA	Migrants in detention centres		x			Potential for transmission in detention centres; healthcare impact
Schotland (Schotland, 2020)	USA	Migrants in detention centres			x		Conditions in detention centres (overcrowding, sanitation)
Mosca (Mosca et al., 2020)	Global	Irregular migrants		x	x		Conditions in detention centres (overcrowding, sanitation), barriers to healthcare (immigration status)
Lenzer (Lenzer, 2020)	USA	Migrants in detention centres		x			Conditions in detention centres (healthcare)
Emelurumonye (Emelurumonye and Miglietta, 2020)	Italy	Migrants in detention centres		x	x		Conditions in detention centres (overcrowding, sanitation, healthcare)
Emelurumonye (Emelurumonye and Miglietta, 2020)	Italy	Migrants in detention centres		x	x		Conditions in detention centres (overcrowding, sanitation, healthcare)
Armitage (Armitage and Nellums, 2020)	Europe	Gypsy, Roma and Traveller population		x	x		Living conditions; barriers to healthcare
Ramírez-Cervantes (Ramírez-Cervantes et al., 2020)	Madrid, Spain	Migrants			x		Socio-economic factors; living conditions (overcrowding)
Valeriani (Valeriani et al., 2020)	Sweden	Migrants		x			Barriers to healthcare (cultural, language)
Giordano (Giordano, 2020)	Belgium	Migrant care workers				x	Occupational risk (frontline, job security)
Kuhlmann (Kuhlmann et al., 2020)	EU	Migrant care workers				x	Occupational risk (frontline, job security)
Kerwin (Kerwin and Warren, 2020)	USA	Migrant workers				x	Occupational risk (frontline/essential, HCWs, job security)
Bureau of Policy & Research (Bureau of Policy and Research 2020)	New York, USA	Migrant workers				x	Occupational risk (frontline)
Haley (Haley et al., 2020)	Canada	Migrant farmworkers		x	x	x	Occupational risk (frontline, job security); overcrowding; healthcare access (immigration status)
Mares (Mares, 2020)	Vermont, USA	Migrant farmworkers		x	x	x	Occupational risk (frontline, job security); overcrowding; healthcare access (immigration status)
Lee (Lee et al., 2020)	USA	Migrant farmworkers		x	x	x	Occupational risk (frontline), overcrowding; healthcare access (cultural, technological)
Chandratre (Chandratre and Soman, 2020)	USA	Migrant physicians				x	Occupational risk (HCWs)
St-Denis (St-Denis, 2020)	Canada	Migrant workers				x	Occupational risk (physical distancing)
Tayaben (Tayaben and Younas, 2020)	Global	Migrant nurses				x	Occupational risk (HCWs)
Nezafat Maldonado (Nezafat Maldonado et al., 2020)	Europe	Migrants		x			Healthcare access (language)
Alahmad (Alahmad et al., 2020)	Kuwait	Migrant workers		x		x	Occupational risk (frontline, job security); healthcare access (structural, cultural, language)
Wong (Wong CL and Chow, 2020)	Hong Kong	South Asian migrants		x			High awareness of pandemic and self-efficacy
Kong (Kong et al., 2020)	Canada	Chinese migrants		x			Healthcare seeking attitudes (Chinese medicine)
Rizzolo (Rizzolo et al., 2020)	USA	Undocumented migrants		x			Co-morbidities (kidney failure, emergency-only haemodialysis)
Orcutt (Orcutt et al., 2020)	Global	Migrants and refugees		x	x		Socio-economic determinants; barriers to healthcare; conditions in camps
Carruthers (Carruthers et al., 2020)	Greece	Refugees and asylum seekers		x	x		Conditions in camps (overcrowding, sanitation, healthcare); healthcare access (immigration status)
Carruthers (Carruthers et al., 2020)	Greece	Refugees and asylum seekers		x	x		Conditions in camps (overcrowding, sanitation, healthcare); healthcare access (immigration status, cultural, language)
Guadagno (Guadagno, 2020)	Global	Migrants		x	x	x	Occupational risk (frontline, job security); healthcare access (immigration status, cultural); overcrowding; conditions in camps and detention centres
Esegbona-Adeigbe (Esegbona-Adeigbe, 2020)	UK	Migrants and asylum seekers		x			Impact on healthcare access
Ali (Ali et al., 2020)	Saudi Arabia	Migrant workers		x	x	x	Occupational risk (frontline); healthcare access (immigration status); overcrowding
OECD (OECD 2020)	OECD	Migrants			x	x	Occupational risk (frontline, job security); overcrowding; socio-economic factors
Turcotte (Turcotte and Savage, 2020)	Canada	Migrants				x	Occupational risk (frontline, HCWs)
Cleveland (Cleveland et al., 2020)	Montreal, Canada	Informants incl. migrants		x	x	x	Socio-economic determinants; occupational risk; overcrowding; barriers to healthcare (language, immigration status)
Gottlieb (Gottlieb et al., 2020)	Germany	Migrants		x	x	x	Socio-economic determinants; occupational risk; overcrowding; barriers to healthcare
Nobody Left Outside (Nobody Left Outside 2020)	Europe	Undocumented migrants		x	x	x	Socio-economic determinants; overcrowding; occupational risk barriers to healthcare

#### 3.3.1 Co-morbidities

Co-morbidities may be a cause of increased COVID-19 risk and/or poor COVID-19 outcomes in migrant populations, but this remains poorly documented. A situational brief reporting on the health or asylum seekers and undocumented migrants in France during COVID-19 concludes they are more likely to have certain chronic conditions that appear to be associated with worse COVID-19 outcomes, such as diabetes mellitus, hypertension, and obesity (Gosselin et al., 2020). In Sweden, a COVID-19 situational report found around 65% of refugees are either overweight or obese compared to 50% in the rest of the population, and around 35% are smokers, which is higher than the general population (Elisabeth et al., 2020). In addition, hospital visits for management of co-morbidities may increase risk of exposure to COVID-19 (Rizzolo et al., 2020). Co-infections may also play a role. In Lisbon, it has been observed that some of the neighbourhoods with increased transmission coincide with areas where TB incidence has been higher (Dias et al., 2020), with over half of patients with TB and COVID-19 in two early case series being migrants (Tadolini et al., 2020, Motta et al., 2020). Migrants in camp settings may be especially vulnerable due to existing illnesses or injuries and prevailing malnutrition and/or poor health in general (Iacobucci, 2020, Hargreaves et al., 2020, Jozaghi and Dahya, 2020, ACAPS 2020).

#### 3.3.2 Healthcare seeking and barriers to care

Testing and treatment for COVID-19 has been made free of charge and exempt from immigration status checks in many countries, with these messages communicated in multiple languages; however, concerns remain that these exemptions do not fully mitigate the extensive barriers that migrants experience in accessing healthcare (Guadagno, 2020, Wood and Devakumar, 2020, Orcutt et al., 2020). Concerns within migrant communities that COVID-19 treatment might be chargeable, or that undocumented migrants might be identified by health systems on presentation remain, and could prevent early presentation and testing in migrants who distrust authorities (Doctors of the World, 2020, Kanlungan Filipino Consortium 2020, Germain and Yong, 2020). In the US, where nearly half of undocumented adult migrants and a quarter of lawfully present adult migrants lack health insurance (Cholera et al., 2020), or have insurance that relies on a specific employer, migrants may avoid seeking care for fear of losing their job and being deported (Doyle, 2020). Various federal policies deter migrants from health seeking (Page et al., 2020, Capps and Gelatt, 2020, Bakhiet et al. 2020, Zelaya et al. 2020). For example, undocumented migrants in the US are ineligible for federally funded healthcare programmes such as Medicare and Medicaid (Wilson et al., 2020), and the ‘public charge’ rule introduced in February 2020 makes migrants who receive a broad range of cash and noncash benefits ineligible to apply for citizenship and residency (Langellier, 2020, Behbahani et al., 2020), deterring treatment-seeking, particularly so in jobs that are often criminalised such as sex work (Lam, 2020). US Immigration and Customs Enforcement (ICE) raids have continued in migrant communities over lockdown, and have further damaged trust and deterred migrants from testing and treatment (Lopez and Holmes, 2020).

In an online survey of undocumented migrants (students who entered as minors) in the US (May 2020), 10% said that they or an immediate family member suspected COVID-19 infection at some point but did not get tested for fear of detainment or deportation, and 1 in 5 said they would be 'extremely worried' for this reason (Goodman et al., 2020).

Healthcare access for migrants and refugees in camp settings can be limited, lacking medical personnel, equipment and pharmaceuticals (Vonen et al., 2020), with poor or absent testing facilities (Medact, 2020). A modelling study has suggested that once the virus enters refugee camps, it can spread quickly, overwhelming hospitals and healthcare facilities (Hernandez-Suarez et al., 2020).

With routine services closed due to the pandemic, concerns have been raised that migrants have struggled to navigate the new systems (Doctors of the World 2020, Valeriani et al., 2020, Esegbona-Adeigbe, 2020, Nobody Left Outside 2020) and it has exacerbated migrants’ exclusion from health services (Desai and Samari, 2020, Santos et al., 2020, Aragona et al., 2020, Clarke et al., 2020, Devillanova et al., 2020). Migrants may experience challenges in accessing healthcare remotely (Doctors of the World 2020, Brickhill-Atkinson and Hauck, 2020, Page et al., 2020, Langellier, 2020, Wernly et al., 2020); however, telemedicine may also offer opportunities that need to be further explored in these populations (Warner et al., 2020, Green et al., 2020).

Migrants often have difficulties understanding public health messaging due to cultural and language barriers (Doctors of the World 2020, Cholera et al., 2020, Bodenmann et al., 2020). Public health guidance in many countries was not initially tailored to the needs of migrant and ethnic minority groups (Doctors of the World 2020, Nezafat Maldonado et al., 2020); in the UK non-governmental organisations (NGOs) translated material into 51 languages to make it more accessible (Patel et al., 2020). In Denmark, a series of qualitative interviews with migrants found that they felt uncertain regarding government guidance for COVID-19; although written material was translated into 19 languages, it was not effectively disseminated (Institut for Menneskerettigheder 2020). In Montreal, Canada, it took two months after lockdown started for the Public Health directorate to publish official multilingual fact sheets on COVID-19 guidelines, and information phone lines only operate in French and English. Those who had arrived most recently, had lower language (French/English) ability or lower literacy had more difficulty accessing local COVID-19 information (Cleveland et al., 2020). In a rapid review to assess communications targeting migrant populations across Council of Europe Member States only 48% (23/47) translated information into at least one foreign language (Nezafat Maldonado et al., 2020). Information on testing or healthcare entitlements in common migrant languages was only found in 6% (3/47) of countries and no government produced risk communications on disease prevention targeting people in refugee camps or informal settlements. Poor language competence was linked to low testing rates in two studies (Guttmann et al., 2020, Kim et al., 2020).

A potential lack of knowledge and awareness of COVID-19 among migrant groups or spread of misinformation has been reported (Doctors of the World 2020). For example, in qualitative interviews conducted in a migrant reception centre in Rocca di Papa, Italy between February and July 2020, there was low awareness of the danger of the pandemic, especially among migrants from sub-Saharan Africa (Ceccarelli et al., 2020). There is some evidence that traditional Chinese medicine may have been used as a means of preventing COVID-19 among Chinese immigrants in Canada (Bodenmann et al., 2020, Kong et al., 2020). Conversely, migrants may be more likely to comply with preventative measures such as mask wearing, especially those migrating from Asian countries where this is more of a cultural norm (Guo et al., 2020, Zhang and Zhao, 2020). A questionnaire among 352 Indian, Pakistani, and Nepalese migrants in Hong Kong found migrants expressed certain misconceptions regarding the prevention of COVID-19 infection, but perceived the risk of disease as mild, had positive attitudes regarding its prevention, and implemented recommended disease-preventive measures (Wong and Chow, 2020).

#### 3.3.3 Camps, detention centres, and overcrowded accommodation

Refugee camps are typically crowded, and are often built quickly and with little regard to such things as tent spacing. In these settings, where social distancing and personal hygiene is difficult, the spread of COVID-19 is facilitated (Greenaway et al., 2020, Iacobucci, 2020, Hargreaves et al., 2020, Jozaghi and Dahya, 2020, ACAPS 2020, Vonen et al., 2020, Hargreaves et al., 2020, Alawa et al., 2020, Peprah, 2020, Spernovasilis et al., 2020, Kondilis et al., 2020, The Lancet 2020, Gilman et al., 2020, Alemi et al., 2020, Carruthers et al., 2020, Carruthers et al., 2020, Veizis, 2020). For example, the Moria camp in Greece had an estimated population density of 133,000 per km^2^, with reports of one water tap shared between 1,300 people in some areas of the camp (ACAPS 2020).

Conditions in detention or reception facilities are similarly conducive to the spread of COVID-19, with confined and poorly ventilated spaces (Doctors of the World 2020, Cross and Benson, 2020, Logar and Leese, 2020, Meyer et al., 2020, Irvine et al., 2020, Schotland, 2020, Mosca et al., 2020). In the US, there have been concerns that ICE facilities have violated their own standards as well as those from the Center for Disease Prevention and Control (CDC), for failing to test sick detainees (Openshaw and Travassos, 2020, Lenzer, 2020). Living conditions in reception facilities in Europe are overcrowded (European Centre for Disease Prevention and Control 2020, Emelurumonye and Miglietta, 2020, Emelurumonye and Miglietta, 2020). Gypsy, Roma and Traveller populations are also at risk due to living in potentially crowded conditions, their nomadic way of life, and reduced engagement with health services (Doctors of the World 2020, Armitage and Nellums, 2020). Many migrant workers live in employer-provided shared accommodation, considered high-risk for COVID-19 (Doyle, 2020, Mares, 2020, Haley et al., 2020, Lee et al., 2020).

Migrants in the community are more likely to live in shared or overcrowded accommodation than non-migrants in host countries (Doctors of the World 2020). 235 (59%) of 399 of patients admitted to a medicalised hotel in Madrid in March to May 2020 were migrants: the main reason for referral was a lack of housing that supported quarantining, for example due to overcrowding, which was correlated with migrant status (*χ^2^*=19.4, *p* < 0.01) (Ramírez-Cervantes et al., 2020). At a clinic in Milan, the proportion of undocumented migrants who were homeless nearly doubled from 8.8% to 17.1% in the months immediately before and during/after lockdown (Devillanova et al., 2020). In a survey of precarious Filipino migrants in the UK, most of whom were undocumented migrants, 58% of respondents lived in shared houses, 1 in 5 were homeless, had no fixed address, or were staying temporarily with friends (on average sharing a bedroom with 1-2 others) (Kanlungan Filipino Consortium 2020).

Across all OECD countries, migrants are more likely to live in sub-standard accommodation (23% versus 19% in the native-born) and twice as likely to live in overcrowded dwellings (17% versus 8%) which could influence transmission and exposure (OECD, 2020). Living in neighbourhoods with higher household density was associated with higher positivity rates for COVID-19 in Ontario, Canada, but especially for migrants (Guttmann et al., 2020). Migrants are also more likely to live in multigenerational houses, with implications for transmission from younger to older and more vulnerable household members (Cholera et al., 2020, Langellier, 2020).

#### 3.3.4 Occupational risk

Migrants are disproportionately represented in front-line public-facing jobs, such as in the fields of healthcare, social work, hospitality, retail, delivery and household services, and in menial jobs that can place them at increased exposure of COVID-19 (Guadagno, 2020, Cross and Benson, 2020, Haley et al., 2020, Giordano, 2020, Kuhlmann et al., 2020, Ali et al., 2020, Gottlieb et al., 2020). On average, 13% of all key workers in the EU are immigrants (Migration Data Portal 2020). Based on 2018 US Census Bureau data for a report on COVID-19 impacts, 69% of all immigrants in the US labour force and 74% of undocumented workers were reported to be essential workers, compared to 65% of the native-born labour force; 70% of refugees and 78% of Black refugees are essential workers (Kerwin and Warren, 2020), with non-US-citizens making up 9% of the labour force but 22% of workers in the agricultural industry, for example (Langellier, 2020). In New York, the hardest hit US city during the first wave of the pandemic, 50% of non-governmental frontline workers are migrants (Bureau of Policy and Research 2020).

Migrants may need to carry on working or risk losing their job (Kanlungan Filipino Consortium 2020, Giordano, 2020). This is especially true for migrants in informal ‘no work, no pay’, with precarious contracts, or exploitative employment, including undocumented migrants who fall outside of government safety nets (Kanlungan Filipino Consortium 2020, Bureau of Policy and Research 2020). A Canadian analysis found that workers in low-income occupations (especially women, migrants, and visible minority groups) are employed in occupations that put them at greater risk of exposure to COVID-19 than other workers; low‐income workers may face financial disincentives for absence even if they are sick or vulnerable, increasing workplace transmission (Armitage and Nellums, 2020). Migrants are also potentially more likely to rely on public transport to get to work, again increasing their possible exposure to COVID-19 infection (Langellier, 2020).

Not all migrants are unskilled or work in low-skilled occupations, however. A substantial proportion of doctors, nurses, and other medical specialists in countries such as Germany, France, US, Canada, and UK are migrants (Migration Data Portal 2020, Chandratre and Soman, 2020). In Canada (2016 data), more than a third of nurse aides, orderlies and patient service associates were migrants, with Black and Filipino women particularly over-represented (Turcotte and Savage, 2020). Data are lacking on the impact of COVID-19 on this occupational group, and on hospital cleaning and maintenance staff who in many EU countries also tend to be migrants. In a Canadian analysis, migrants in health occupations were found to have a slightly higher mean occupational risk of exposure to diseases/infections such as COVID-19 than Canadian-born workers (St-Denis, 2020). Employment as a healthcare worker in Ontario accounted for a disproportionate number of COVID-19 cases among migrants, especially women (Guttmann et al., 2020). Concerns have also been raised about inadequate access to or use of PPE, overrepresentation of migrants in low paying paramedical roles, or difficulties in self-isolating because of staff shortages at the start of the pandemic (Greenaway et al., 2020, Tayaben and Younas, 2020).

Living in low-income neighbourhoods was strongly correlated with test positivity for newly-arrived migrants but not for Canadian-born and long-term residents (Guttmann et al., 2020). In addition, the association between percentage of immigrants living in a given area of Ontario and diagnoses of COVID-19 is attenuated when adjusting for covariates such as household income, educational attainment, and household density (Sundaram et al., 2020). In Swedish data, socioeconomic status (including disposable income and employment status), number of working age household members and neighbourhood population density attenuated up to half of the increased COVID-19 mortality risk, but not all-cause mortality (Rostila et al., 2020), indicating that these factors play a role but cannot account entirely for the observed disparity.

## 4 Discussion

This systematic review is the first attempt to bring together global datasets on the impact of COVID-19 on migrants, and to assess the critical risk factors and vulnerabilities involved, in what is a rapidly evolving field. We found that migrants are at high risk of COVID-19 infection and over-represented in confirmed COVID-19 cases, with data suggesting an elevated risk for COVID-19 among undocumented migrants, migrant health and care workers, and migrants housed in camps and labour compounds. Available data point to a similarly disproportionate representation of migrants in reported COVID-19 deaths, as well as increased all-cause mortality in migrants in reporting countries in 2020, though data are limited. In general, migrants were found to have higher levels of many of the risk factors and vulnerabilities for COVID-19, as a result of increased exposure due to high-risk or precarious occupations, overcrowded accommodation, legal-administrative barriers to healthcare services and low levels of language competence, all of which have a potentially negative impact on awareness of the problem and/or ability to take remedial action – including testing uptake and activities to reduce exposure. These data are of immediate relevance to national public health responses, be it in terms of policies or programmatic actions tailored to reach migrants.

In the most recent and largest systematic review of 18,728,893 patients in datasets reporting clinical outcomes for COVID-19 by ethnicity (42 studies from the US, 8 from the UK to 31 Aug), authors report an increased risk of infection in Black and Asian ethnicities (Asian pooled adjusted RR 1.50 [95% CI 1.24–1.93]; Black 2.02 [1.67–2.45]) compared to White individuals, with Asian individuals being at higher risk of hospital admission to intensive care and risk of death, even after adjusting for confounders such as age, sex, and co-morbidities (Sze S and Nevill, 2020). Other research has highlighted high seroprevalence rates for COVID-19 in people living in precarious situations, suggesting over-exposure of marginalised groups (Roederer et al., 2020). These datasets will include migrants as a subpopulation, but do not disaggregate by migrant status. Our analysis suggests that migrants specifically have an increased risk of infection and points to striking increases in all-cause mortality data among certain migrant groups in the few countries that have reported on this in 2020. More robust data on cases, testing uptake, hospitalisations and deaths from COVID-19 among migrants is therefore warranted and considered urgent. There is also a need to strengthen data systems in HICs so as to better understand the distribution of particular health outcomes in migrant populations, not only with respect to COVID-19 but in other disease areas as well.

In this analysis, we report data that defines a unique set of risk factors and vulnerabilities experienced by migrants in HICs that are influencing exposure and outcomes to COVID-19. These risk factors and vulnerabilities are, in large part, related to their health and social situation in the host country, and the barriers to accessing health systems (including preventative testing and treatment) that they face, which have been well reported for other infectious diseases (Noori et al., 2020). Risk factors include lower levels of language proficiency rendering public health messaging inaccessible. Low host country language competence, which is particularly the case with more recently arrived migrants, was seen to be associated with lower rates of testing in two studies, but higher rates of positivity when tested (Guttmann et al., 2020, Kim et al., 2020). We know that few countries specifically targeted public health messaging to migrants, which could have resulted in their exclusion from the larger public health response (Nezafat Maldonado et al., 2020). Precarious occupations and social situations mean that public health proposals such as work-from-home, self-isolation, avoidance of public transport, and rapid testing uptake are not relevant for many migrants and point to a type and degree of exclusion or restricted access to mainstream health systems.

Tens of thousands of migrants in HICs are excluded or restricted from accessing mainstream health systems because of their immigration status, likely a major barrier to accessing testing and treatment, and eventual vaccine roll out. Previous data for other infections has shown migrants may be late presenters to health services (Potter et al., 2020), presenting only where necessary due to concerns around immigration and lack of trust, lack of knowledge of the health system, and barriers to registration and access. These findings strongly support arguments for more tailored and targeted public health initiatives to these groups, including information and communication around testing, contact tracing, isolation, and when to present, as well as tackling misinformation. Actions on behalf of migrants should be undertaken with and through trusted community channels, and developed through direct engagement with at-risk migrant groups. Several groups have called for the temporary suspension of policies that exclude migrants from health systems during the pandemic (Orcutt et al., 2020), something several countries have done, along with stressing the importance of inclusion of these groups in ongoing protective measures, information campaigns and health services provision ((The Migrant and Ethnic Health Section of the European Public Health Association (EUPHA) 2020, The Migrant and Ethnic Health Section of the European Public Health Association (EUPHA) 2020)). WHO and other agencies have reinforced the need to ensure migrants in camps and closed facilities are offered screening, triage testing, and provided with care (Chandratre and Soman, 2020). UN agencies have also stressed the human rights of refugees and migrants and the need to ensure that COVID-19 responses respect these rights (UNHCR, IOM, OHCHR, WHO 2020).

This review has some inevitable limitations. It was not possible to engage an expert in every HIC and as a result some national statistics and grey literature may have been missed. However, we engaged widely through our international networks to source local literature, and the WHO database sources both peer-reviewed, pre-prints, and grey literature from a diverse range of databases that would not normally have been searched individually for a systematic review. We are therefore confident that we have included the majority of datasets available to 18 Nov. In addition, we have included non-peer reviewed grey literature and pre-prints in the narrative synthesis with obvious limitations. Due to the rapidly evolving nature of the pandemic we felt this was justified and strengthens our description of the current situation facing migrants in HICs. In Table 1 and Supplementary Table 1 we have clearly stated all data sources and have given a quality appraisal score to them.

Panel 1 sets out some of the implications of this work for further research and for health policies. Understanding the lived experience of marginalised migrants will be vital to tackling issues around barriers to care (including of migrants with long-term symptoms), testing uptake, and obstacles and facilitators to eventual COVID-19 vaccination and ensuring good vaccine coverage of, and uptake by migrants and ethnic minorities (Paul et al., 2020). We believe our findings are of immediate relevance to the ongoing public health responses and should inform policies seeking to minimise exposure to COVID-19 in migrants and ensure their inclusion, through innovative and nuanced solutions with community engagement at their centre.

**Panel 1: Further research and next steps**

**Table:** 

**Strengthen data collection and future planning** • Initiate large retrospective and prospective studies, disaggregating by migrant status, exploring disparities in testing and diagnosis, hospitalisations, and COVID-19-related deaths in migrants • Collate and conduct ongoing analysis of data on COVID-19 vaccine uptake by migrants when vaccine roll out starts, to identify disparities early on so they can be addressed • Ensure more consistent and complete incorporation of migrant status in surveillance and health information systems taking into account gender, ethnic, linguistic, educational and occupational diversity in migrant populations • Create more empirical evidence on the link between risk factors identified in migrants and the role they play in driving disparities in clinical outcomes • Development of pandemic preparedness plans that address migration and migrants, and can be shared by countries **Delivery of more effective public health messaging** • Co-produce carefully researched messaging on COVID-19 prevention, testing and treatment, contact tracing, and self-isolation with affected communities, tailored to different cultural and social realities and that considers the unique risk factors and vulnerabilities of migrant populations and offers them meaningful solutions and support mechanisms to reduce their exposure • Ensure rapid quality translation and more effective dissemination of public health messaging and directives into common migrant languages • Engagement of diverse high-risk migrant communities, through localised support and community champions, in defining how best to deliver credible information and support on COVID-19 testing, reducing their exposure, social support, and facilitating vaccine roll out, alongside exploring mechanisms to build trust in health systems and tackle misinformation **Better consider specific migrant groups** • Proactively include extremely marginalised migrants living in camps, reception centres, detention centres, labour compounds, and undocumented migrants and others facing known structural barriers to health systems in the COVID-19 response **Long-term approaches to tackling disparities facing migrants in HICs** • Facilitate more inclusive and culturally competent health systems, now and beyond this pandemic • Develop evidence-based inter-sectoral policies and strategies designed to improve the overall health and social conditions of migrants and respect the rights of migrants to basic human security in host countries • Facilitate meaningful change to support the inclusion of migrants in host health systems, in alignment with the principles of Universal Health Coverage and the UN Sustainable Development Goals to leave no-one behind

## Contributions

SH conceived the idea for the review and ran the literature searches. SEH, SH, and CC did the abstract and full text screening, data extraction, and synthesis. AD and CC did the quality appraisal. AFC, MO, MN, AR, CG, KB, AV, FW, IC-M, FS, and BN provided national datasets and grey literature. SEH and SH wrote a first draft of the paper with input from all authors.

## Funding

NIHR; Academy of Medical Sciences.

## Conflicts of Interest

The authors report no conflicts of interest to declare

## Figures and Tables

**Table:** 

1. Andorra	36. Isle of Man	73. St Martin (French part)
2. Antigua and Barbuda	37. Israel	74. Sweden
3. Aruba	38. Italy	75. Switzerland
4. Australia	39. Japan	76. Trinidad and Tobago
5. Austria	40. Korea, Rep.	77. Turks and Caicos Islands
6. The Bahamas	41. Kuwait	78. United Arab Emirates
7. Bahrain	42. Latvia	79. United Kingdom
8. Barbados	43. Liechtenstein	80. United States
9. Belgium	44. Lithuania	81. Uruguay
10. Bermuda	45. Luxembourg	82. Virgin Island
11. British Virgin Islands	46. Macao SAR, China	
12. Brunei Darussalam	47. Malta	
13. Canada	48. Mauritius	
14. Cayman Islands	49. Monaco	
15. Channel Islands	50. Nauru	
16. Chile	51. Netherlands	
17. Croatia	52. New Caledonia	
18. Curacao	53. New Zealand	
19. Cyprus	54. Northern Mariana Islands	
20. Czech Republic	55. Norway	
21. Denmark	56. Oman	
22. Estonia	57. Palau	
23. Faroe Islands	58. Panama	
24. Finland	59. Poland	
25. France	60. Portugal	
26. French Polynesia	61. Puerto Rico	
27. Germany	62. Qatar	
28. Gibraltar	63. Romania	
29. Greece	64. San Marino	
30. Greenland	65. Saudi Arabia	
31. Guam	66. Seychelles	
32. Hong Kong SAR, China	67. Singapore	
33. Hungary	68. Sint Maarten (Dutch part)	
34. Iceland	69. Slovak Republic	
35. Ireland	70. Slovenia	
	71. Spain	
	72. St Kitts and Nevis	

## Appendix 1: Search Strategy

TI:(Ancest* OR Diaspor* OR ethnic* OR Ethnoc* OR Ethnog* OR "Identity politics" OR Ingroups OR  outgroups OR Intersectionality OR Kinship OR "Minority group*"~3 OR "minority population*"~2  OR minorities OR Multicultu* OR Polyethnic* OR "Population genetics" OR Race OR races OR racial OR Tribe* OR latino*) OR AB:(Ancest* OR Diaspor* OR ethnic* OR Ethnoc* OR Ethnog* OR "Identity politics" OR Ingroups OR Outgroups OR Intersectionality OR Kinship OR "Minority group*"~3 OR "minority population*"~2  OR minorities OR Multicultu* OR Polyethnic* OR "Population genetics" OR Race OR races OR racial OR Tribe* OR latino*) OR "afro american*"~3 OR BAME OR latino* OR roma OR romani OR refugee* OR  immigrant* OR “migrant” OR "displaced person" OR "displaced persons"  OR "social determinant*"~2 OR "latin population" OR "latin group*" OR "people of color" OR "people of colour"

## Appendix C Supplementary materials

Image, application 1
