# Supplementary material for: Clinical outcomes and risk factors for COVID-19 among migrant populations in high-income countries: A systematic review
Source: J Migr Health. 2021 Apr 22;3:100041. doi: 10.1016/j.jmh.2021.100041 (PMC8061095; doi:10.1016/j.jmh.2021.100041)
Supplement: Supplementary file 1 [file mmc1.docx]

**Appendix 1: Search Strategy**

TI:(Ancest* OR Diaspor* OR ethnic* OR Ethnoc* OR Ethnog* OR "Identity politics" OR Ingroups OR  outgroups OR Intersectionality OR Kinship OR "Minority group*"~3 OR "minority population*"~2  OR minorities OR Multicultu* OR Polyethnic* OR "Population genetics" OR Race OR races OR racial OR Tribe* OR latino*) OR AB:(Ancest* OR Diaspor* OR ethnic* OR Ethnoc* OR Ethnog* OR "Identity politics" OR Ingroups OR Outgroups OR Intersectionality OR Kinship OR "Minority group*"~3 OR "minority population*"~2  OR minorities OR Multicultu* OR Polyethnic* OR "Population genetics" OR Race OR races OR racial OR Tribe* OR latino*) OR "afro american*"~3 OR BAME OR latino* OR roma OR romani OR refugee* OR  immigrant* OR “migrant” OR "displaced person" OR "displaced persons"  OR "social determinant*"~2 OR "latin population" OR "latin group*" OR "people of color" OR "people of colour"

**Appendix 2: World Bank High-Income Countries (2020)**

1. Andorra
2. Antigua and Barbuda
3. Aruba
4. Australia
5. Austria
6. The Bahamas
7. Bahrain
8. Barbados
9. Belgium
10. Bermuda
11. British Virgin Islands
12. Brunei Darussalam
13. Canada
14. Cayman Islands
15. Channel Islands
16. Chile
17. Croatia
18. Curacao
19. Cyprus
20. Czech Republic
21. Denmark
22. Estonia
23. Faroe Islands
24. Finland
25. France
26. French Polynesia
27. Germany
28. Gibraltar
29. Greece
30. Greenland
31. Guam
32. Hong Kong SAR, China
33. Hungary
34. Iceland
35. Ireland
36. Isle of Man
37. Israel
38. Italy
39. Japan
40. Korea, Rep.
41. Kuwait
42. Latvia
43. Liechtenstein
44. Lithuania
45. Luxembourg
46. Macao SAR, China
47. Malta
48. Mauritius
49. Monaco
50. Nauru
51. Netherlands
52. New Caledonia
53. New Zealand
54. Northern Mariana Islands
55. Norway
56. Oman
57. Palau
58. Panama
59. Poland
60. Portugal
61. Puerto Rico
62. Qatar
63. Romania
64. San Marino
65. Saudi Arabia
66. Seychelles
67. Singapore
68. Sint Maarten (Dutch part)
69. Slovak Republic
70. Slovenia
71. Spain
72. St Kitts and Nevis
73. St Martin (French part)
74. Sweden
75. Switzerland
76. Trinidad and Tobago
77. Turks and Caicos Islands
78. United Arab Emirates
79. United Kingdom
80. United States
81. Uruguay
82. Virgin Islands
